# A realistic MR compatible aortic phantom to validate hemodynamic parameters from MRI data: aortic coarctation patients comparison using catheterization

**DOI:** 10.1186/1532-429X-17-S1-P199

**Published:** 2015-02-03

**Authors:** Jesus Urbina, Julio Sotelo, Cristian Tejos, Pablo Irarrazaval, Marcelo E Andia, Reza Razavi, Israel Valverde, Sergio Uribe

**Affiliations:** Biomedical Imaging Center, Pontificia Universidad Católica de Chile, Santiago, Chile; Imaging Sciences and Biomedical Engineering, King’s College London, London, UK; Institute of Biomedicine of Seville, Universidad de Sevilla, Seville, Spain; School of Medicine, Pontificia Universidad Católica de Chile, Santiago, Chile; Electrical Engineering Department, Pontificia Universidad Católica de Chile, Santiago, Chile; Radiology Department, Pontificia Universidad Católica de Chile, Santiago, Chile; Cardiology Unit, Hospital Virgen del Rocío, Universidad de Sevilla, Seville, Spain

## Background

Recently, 3D printing technologies have emerged as a very innovative technique to produce anatomical replicas. Nevertheless, vessel phantoms built up to now are simplified models, with difficulties to obtain parameters with physiological values. The aim of this work is to show and validate a MR compatible thoracic aorta system, designed to obtain hemodynamic parameters within a range comparable to healthy volunteers and patients with aortic coarctation (AoCo).

## Methods

The phantom is a closed circuit integrated by a MR compatible unit pump with a control unit (Shelley Medical Imaging Technologies) and a realistic aortic model built with flexible silicone (Elastrat). Three non-return valves were installed to avoid negative pressures. Additionally, shut-off valves were added to regulate the flow distribution between the different vessels. We built an 11 mm AoCo, which was placed in the descending aorta just after the left subclavian artery. Additionally, we equipped the system with a catheterization unit to measure invasive pressure along the AoCo.

**Figure 1:**
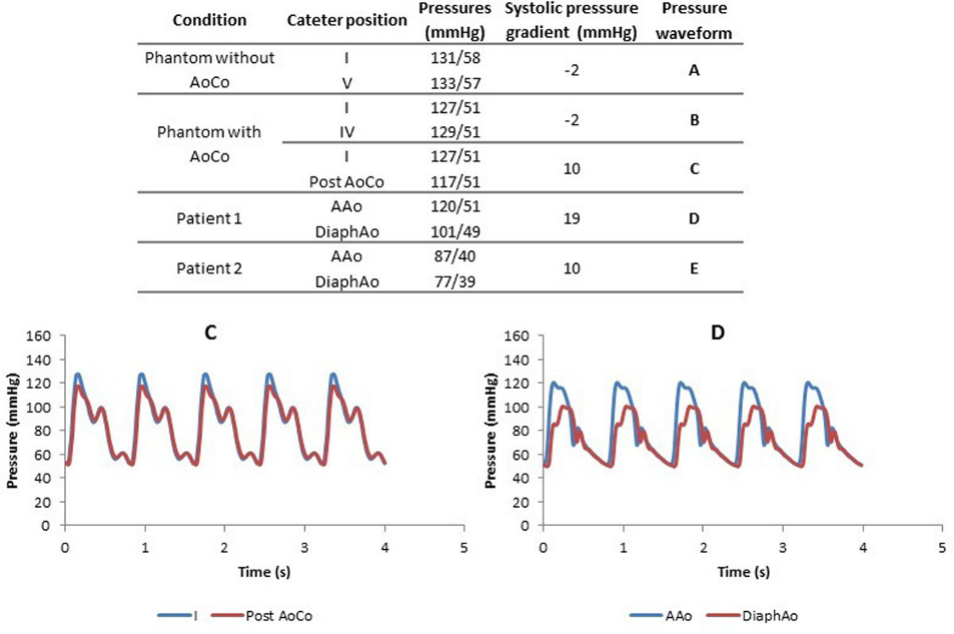
**Pressure values are represented from the phantom without and with the AoCo and AoCo patient using catheterization.** The curve C represents the pressure measured in the phantom with AoCo between the positions I and post AoCo. D represents the pressure measured between the ascending aorta (AAo) and the diaphragmatic aorta (DiaphAo) of the patient 1.

Experiments were performed on a 1.5 T MR-system (Philips). Two different situations were studies: a phantom without and with an 11 mm AoCo. For each situation we acquired 2D and 3D PC-MRI data. The 2D PC-MRI data were acquired in 5 section of the aorta (figure 1) and the 3D PC-MRI data were acquired in the entire phantom. Velocity and flow related parameters were obtained from both methods using the commercial software GTFlow 2.0.10 (Gyrotools LCC). We measured the systolic pressure gradient with the catheterization system.

Phantom data were compared with 3D PC-MRI data acquired in 10 healthy volunteers, and 2 unrepaired aortic coarctation patients. Patient data were acquired in a combined MRI/Catheter interventional suite.

## Results

**Figure 2:**
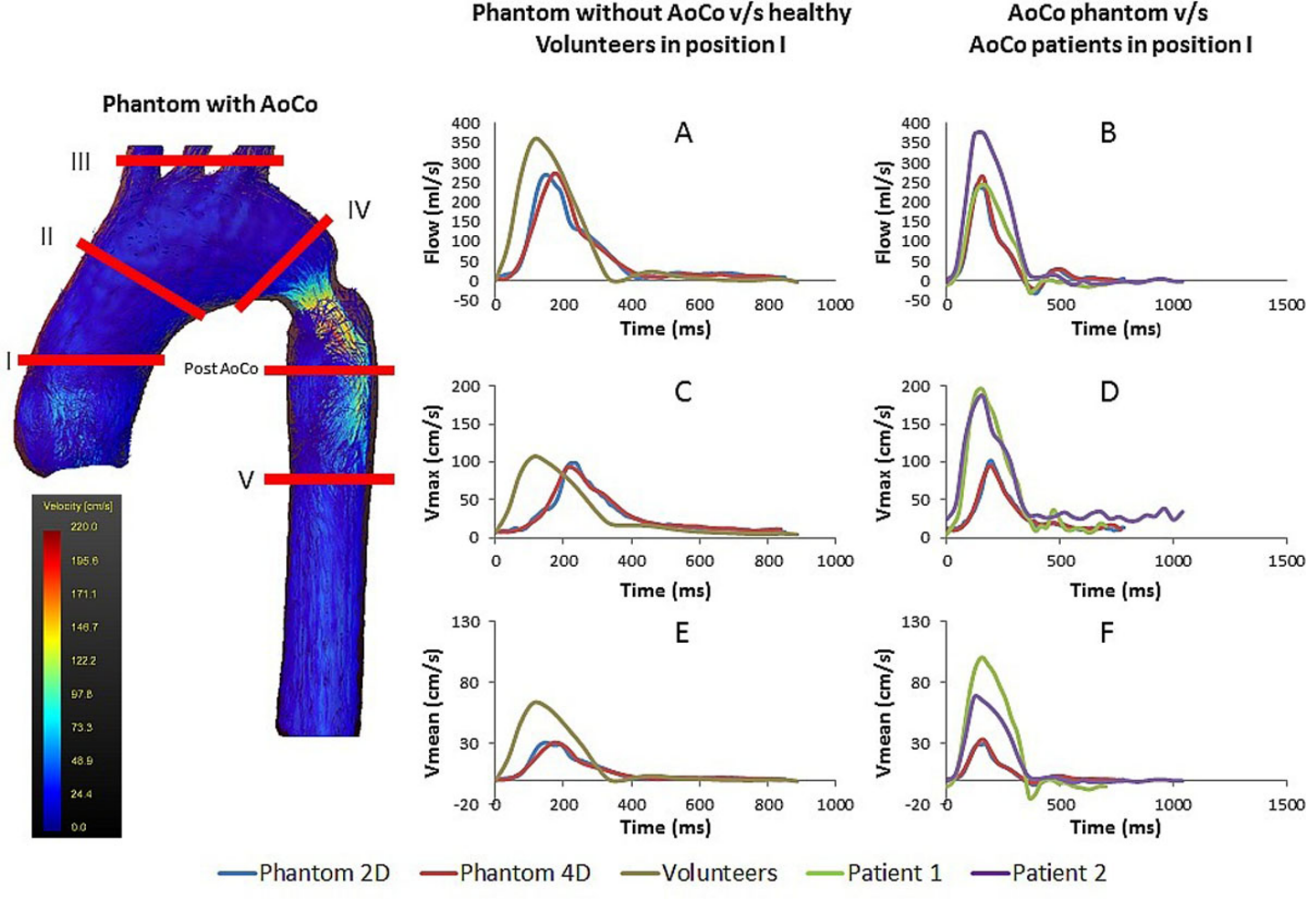
**Velocity patterns (pathlines) of the phantom with AoCo and 2D position along the aorta are represented in the left side.** In the right side, peak flow (A and B), maximum velocities (C and D) and mean velocities (E and F) curves in the position I from the phantom, volunteers and patients are represented. Phantom curves were built from 2D and 3D PC-MRI data.

We obtained realistic hemodynamic parameters, similar to volunteers and patients values. Using 4D flow data in the position I (Ascending aorta), the aortic phantom without AoCo had a peak flow and velocity of 270 ml/s and 91 cm/s and volunteers of 357 ml/s and 107 cm/s respectively. Phantom with AoCo had a peak flow and velocity of 265 ml/s and 95 cm/s, patients 1 of 244 ml/s and 196 cm/s and patient 2 of 376 ml/s and 187 cm/s respectively (Figure 1). The CO in the phantom without AoCo and volunteers were 3.5 and 4.5 L/min. The CO in the phantom with AoCo and patients 1 and 2 were 3.0, 3.9 and 5.7 L/min. Phantom pressure without and with the AoCo were 131/58 and 127/51 mmHg with a trans-coarctation systolic pressure gradient of 10 mmHg. Patients 1 had a pressure of 120/51 mmHg with a gradient of 20 mmHg. Patient 2 had a pressure of 87/39 mmHg with a gradient of 11 mmHg (Figure 2). Flow, velocity and pressure waveforms in the phantom without and with AoCo were similar to healthy volunteers and patients respectively.

## Conclusions

Results in this study demonstrate the feasibility of our realistic aortic system to simulate physiologic and pathologic hemodynamic parameters through an AoCo.

## Funding

### Grant Sponsor

Fondo Nacional de Desarrollo Científico y Tecnológico (FONDECYT), Ministerio de Educación, Chile. Grant Number: FONDECYT #1141036.

